# Pharmaco-Magnetic Resonance as a Tool for Monitoring the Medication-Related Effects in the Brain May Provide Potential Biomarkers for Psychotic Disorders

**DOI:** 10.3390/ijms22179309

**Published:** 2021-08-27

**Authors:** Katrin Aryutova, Drozdstoy Stoyanov

**Affiliations:** Department of Psychiatry and Medical Psychology, Research Institute, Medical University Plovdiv, 4002 Plovdiv, Bulgaria; katrin.aryutova@phd.mu-plovdiv.bg

**Keywords:** schizophrenia, psychosis, cognitive symptoms, neurotransmission, dopamine, glutamate, brain connectivity, pharmacological magnetic resonance imaging

## Abstract

The neurodegenerative and neurodevelopmental hypotheses represent the basic etiological framework for the origin of schizophrenia. Additionally, the dopamine hypothesis, adopted more than two decades ago, has repeatedly asserted the position of dopamine as a pathobiochemical substrate through the action of psychostimulants and neuroleptics on the mesolimbic and mesocortical systems, giving insight into the origin of positive and negative schizophrenic symptoms. Meanwhile, cognitive impairments in schizophrenia remain incompletely understood but are thought to be present during all stages of the disease, as well as in the prodromal, interictal and residual phases. On the other hand, observations on the effects of NMDA antagonists, such as ketamine and phencyclidine, reveal that hypoglutamatergic neurotransmission causes not only positive and negative but also cognitive schizophrenic symptoms. This review aims to summarize the different hypotheses about the origin of psychoses and to identify the optimal neuroimaging method that can serve to unite them in an integral etiological framework. We systematically searched Google scholar (with no concern to the date published) to identify studies investigating the etiology of schizophrenia, with a focus on impaired central neurotransmission. The complex interaction between the dopamine and glutamate neurotransmitter systems provides the long-needed etiological concept, which combines the neurodegenerative hypothesis with the hypothesis of impaired neurodevelopment in schizophrenia. Pharmaco-magnetic resonance imaging is a neuroimaging method that can provide a translation of scientific knowledge about the neural networks and the disruptions in and between different brain regions, into clinically applicable and effective therapeutic results in the management of severe psychotic disorders.

## 1. Introduction

Psychosis is not a nosological entity, but rather a clinical condition consisting of numerous symptoms that may be a common clinical outcome of a variety of causes. While the concept and definition of psychosis is defined by the core clinical symptoms of delusions, hallucinations, and disorganized thinking, these symptoms are most likely the common final consequences of a variety of different etiopathogenetic pathways, which may all lead to an analogous clinical picture [1].

Schizophrenia and other psychotic disorders are a heterogeneous group of mental illnesses that are frequently categorized together for practical reasons. However, schizophrenia is the most prevalent, debilitating, and socially significant disorder among this group [2]. Schizophrenia is distinguished by a wide range of psychopathological features: positive symptoms (psychotic symptoms: delusions, hallucinations and disorganized behavior), negative symptoms (avolition, alogia, autism, affect flattening, social disengagement, etc.), and cognitive impairment (attention, memory and executive functions deficits) [3]. Positive symptoms usually resolve and relapse, although many individuals maintain protracted psychotic symptoms. Negative and cognitive symptoms are often persistent and are correlated with long-term consequences on social function [4]. The first episode of psychosis generally occurs in late adolescence or early adulthood, although it is commonly precipitated by a prodromal stage [5] and premorbid cognitive deficits [4].

The diagnosis of schizophrenia is purely clinical, most often made after the first manifestation of psychotic symptoms [6]. In practice, the criteria of the Diagnostic and Statistical Manual of Mental Disorders–5th edition (DSM-V) [7] and the International classification of diseases–10th division (ICD-10) [8] are the “gold standard” used for diagnostic purposes. DSM-V and ICD-10 are guidelines with high reliability [9], as they are applied worldwide by clinicians. However, these manuals, as well as the available psychometric tools (questionnaires, assessment/self-assessment scales), are considered unconventional diagnostic methods for the standard medical framework [10]. This is due to the lack of evidence-based etiological explanations for schizophrenia and other psychotic disorders [11]. Psychiatrists are the only medical professionals who do not examine the organ they are treating, but instead rely on observations of behavior, reported complaints from the patient and/or third parties, and their willingness to draw conclusions about their patients’ personal experiences [12]. This inevitably compromises both the diagnostic and therapeutic process, which may be at the root of the treatment failures that are a common obstacle among schizophrenic patients.

Despite all the described shortcomings, psychiatry is currently in its “heyday” due to the variety of neuroimaging techniques that offer opportunities to study structural and functional aberrations in the central nervous system (CNS). Combining psychopharmacology with functional magnetic resonance imaging (fMRI) in the study of central psychopharmacological mechanisms, could be a successful translational strategy in the search for biomarkers for the validity of psychiatric diagnosis and treatment monitoring in clinical practice.

This review will examine the etiological theories of schizophrenia, the current methods for identifying biomarkers by neuroimaging tools, the possibilities of incorporating research findings into clinical practice, and the potential benefits of applying interdisciplinary efforts in the management of schizophrenia, emphasizing translational neuroscience. We will visualize our desire to combine psychopathological, psychopharmacological and neuroimaging techniques with the ultimate goal of finding the optimal therapeutic method that could successfully treat not only the positive but also the negative and cognitive symptoms that cause severe disability among schizophrenic patients.

## 2. Methods

Studies were searched via Google Scholar and PubMed databases without taking into account the date of publication. Potential candidate studies were identified first using the following search word combinations: etiological theories-relevant (schizophrenia, neurodevelopment hypothesis, neurodegenerative hypothesis, biochemical, neurotransmission, dysconnectivity hypothesis), and MRI-relevant (fMRI, blood-oxygen-level-dependent (BOLD), functional imaging, connectivity, rest, or resting, task, pharmacological MRI). Reference lists of retrieved studies were also searched manually to find additional potential studies of relevance to match the initial keywords set.

## 3. Results

### 3.1. Etiological Theories of Schizophrenia

Various theories on the origins of schizophrenia have been proposed in the past, including the supernatural, somatogenic, and psychogenic theories. According to the supernatural theory, psychotic phenomena are attributed to spells, sins, and the soul’s possession by evil and demonic spirits. According to the somatogenic theory, behavioral disturbances arise as a result of somatic disease, genetic anomalies, brain injury, or metabolic imbalance. Psychogenic theory is concerned with psychotraumatic or stressful events that result in maladaptive behavior [13].

In modern times, with the understanding of the basic and specific biological mechanisms in the CNS, several etiological directions have been formed, which are supported by the relevant genetic, clinical, neuroimaging and especially psychopharmacological observations on the course of psychotic disorders.

#### 3.1.1. Neurodevelopment vs. Neurodegeneration

Since Bender [14] coined the term “developmental encephalopathy” in 1947 to describe schizophrenia, numerous studies have revealed strong links between delayed motor development in children and the onset of the illness [15,16,17]. This finding might indicate aberrant functional development of the cortical-subcortical brain circuits, which is a predictor of future behavioral disorders (in later childhood and adolescence) [18] as well as an inability to organize the brain structure and function properly in adulthood [19,20]. Longitudinal studies have reported that people with schizophrenia develop cognitive impairment, especially in executive functions, even before the full clinical manifestation of psychosis [21,22,23]. These findings support the view that neurodevelopmental abnormalities play a central role in the etiopathogenesis of schizophrenia [24].

Numerous longitudinal studies, on the other hand, have discovered a progressive decrease in cortical thickness over the course of the disease, which correlates with the affected individuals’ ongoing impairment in cognitive and social functioning [25,26,27]. This concept underpins the neurodegenerative hypothesis, which states that schizophrenia is caused by an organic neurodegenerative process that manifests itself in an individual’s behavior through the course of the illness and is most active in the early stages of the disease [28]. However, an obstacle to the acceptance of the neurodegenerative hypothesis is antipsychotic medications, which exhibit a significant effect on brain structure and function of schizophrenic patients. In order to avoid this confounding factor, Lin et al. [29] used MRI to measure the cortical thickness in first-episode and treatment-naive patients with schizophrenia and discovered that the parietal, frontal, temporal, and cingulate zones exhibited a significant age-related reduction of cortical thickness compared to healthy controls, which supports the neurodegenerative hypothesis.

Some scientists propose a conceptual model that consists of a set of pathological mechanisms that occur during the stages of neurodevelopment, as well as later emerging (during the first psychotic episode) brain-degenerative processes, which, when combined with the same causal factors, eventually at some point lead to the clinical manifestation of schizophrenia [30].

#### 3.1.2. Biochemical Explanation of Psychosis

In the second half of the twentieth century, the focus shifted from psychogenic to neurobiochemical theory, thanks to the advent of neuroleptics. Their action on dopamine D2-receptors in mesolimbic and mesocortical structures underlies the “dopamine hypothesis” [31] for schizophrenia, rejecting the psychodynamic “schizophrenogenic mother” [32,33]. The realization that schizophrenic psychosis is not a consequence of a wrong model of child rearing, but a true neurochemical disorder caused by a dysfunction in central dopaminergic neurotransmission [31,34], completely changed the understanding of the origin of psychosis.

The classical dopamine hypothesis for schizophrenia suggests that hyperdopaminergia in the mesolimbic system causes psychotic symptoms [35] and hypodopaminergia in the mesocortical pathway is the reason for negative symptoms. This hypothesis is supported by the correlation between the action of antipsychotic drugs and their efficacy in blocking dopamine D2 receptors [36], as well as by the psychotic phenomena triggered by the dopamine agonists [37]. Studies with amphetamine (psychomimetic drug) in untreated patients with schizophrenia have shown hyperdopaminergic activity in the striatum. Using single-photon emission computed tomography (SPECT) neuroimaging, amphetamine-induced hyperdopaminergia was found to be significantly higher in patients with schizophrenia compared to healthy controls [38,39,40,41]. The same abnormal effect has been found in patients in their first psychotic episode who had never taken antipsychotics [39]. In addition, this hypothesis is supported by clinical and research observations of epileptic seizures caused by lesions in the limbic regions that lead to florid psychotic production [42], as well as observations on individuals with tumors in the limbic structures [43].

**Figure 1:**
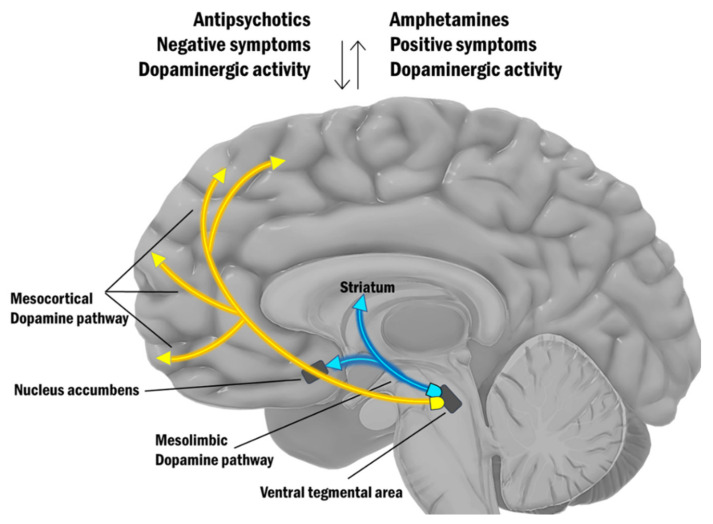
The dopamine hypothesis for schizophrenia.

The classical dopamine hypothesis is presented on Figure 1.

Although the dopamine hypothesis has contributed significantly to the understanding of the clinical effects of psychostimulants, as well as to the introduction of many molecules with antipsychotic effects, it has nevertheless led to certain limitations [44]. For example, the perception of the dopamine hypothesis as an absolute etiological framework limits investigations in neurobiology, shifting researchers’ focus mainly to investigate brain areas with rich dopaminergic neurotransmission and somewhat ignoring findings in other potentially significant areas in the brain. The disturbances of the dopaminergic neurotransmission and the therapeutic efficacy of D2-antagonists are insufficient for a systematic understanding of the complex psychopathology of schizophrenia. Although it provides a satisfactory explanation for the genesis of psychotic symptoms, the disadvantage of the dopamine hypothesis is its inability to explain cognitive and partly negative schizophrenic symptoms, which are responsible for the significant deterioration in schizophrenic patients’ ability to work and social disengagement [45].

**Figure 2:**
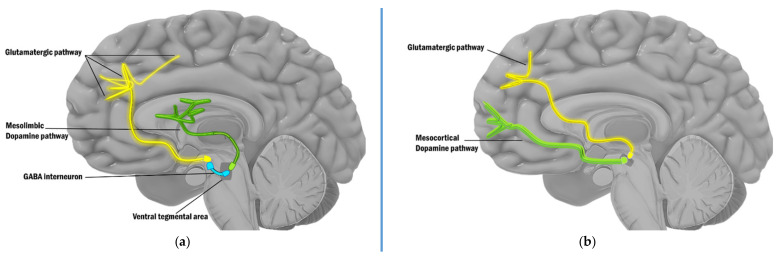
Dopamine pathways’ regulation by the glutamate pathways (green—dopaminergic; yellow—glutamatergic; blue—GABA-ergic): (**a**) Descending glutamatergic pathway connect to the dopaminergic mesolimbic system via a GABA interneuron between them. In the case of disruption, psychotic symptoms emerge; (**b**) The descending glutamatergic pathway exhibits excitatory influence on the mesocortical dopamine pathway, and, in the case of disruption, cognitive deficits emerge.

About four decades ago, an alternative etiological formulation for schizophrenia was postulated based on the action of “dissociative anesthetics,” a class of psychomimetic drugs that includes phencyclidine (PCP) and ketamine. These substances are glutamatergic antagonists that act by blocking the glutamate receptor of the *N*-methyl-*D*-aspartate (NMDA) type [44]. Unlike dopamine agonists (amphetamines), NMDA antagonists such as PCP and ketamine induce positive, negative, and cognitive symptoms that are virtually indistinguishable from those seen in schizophrenia [46,47,48,49]. In addition, it has been suggested that impaired dopaminergic neurotransmission in schizophrenia may itself be secondary to abnormal NMDA-receptor neurotransmission [50,51]. Amphetamine-induced central hyperdopaminergic activity in schizophrenia has already been shown to result from disturbances in the glutamatergic neuronal systems that regulate dopaminergic cellular activity [41]. In addition, abnormalities of glutamatergic afferent neurons from the prefrontal cortex (PFC) to the dopaminergic subcortical areas of the midbrain are likely to be associated with this abnormal regulation, given the evidence of deficiencies in PFC function in schizophrenia [52,53,54,55]. In this sense, dopamine is regulated by cortical glutamate in two ways: as a direct excitator and as an indirect inhibitor. Typically, in healthy individuals, the descending glutamatergic pathway exhibits excitatory influence on the mesocortical dopamine pathway, guiding higher brain regions in the cortex. Dopamine misfiring may lead to cognitive impairments and symptoms of schizophrenia if NMDA receptors in the midbrain are malfunctioning. The glutamate neurons that connect to the dopaminergic neurons in the limbic system, on the other hand, have a gamma-aminobutyric acid (GABA) interneuron between them. GABA acts as a brake by inhibiting the release of dopamine. Excess dopamine can contribute to the occurrence of positive symptoms of psychosis if this break is removed, for example, by less glutamatergic activity (Figure 2).

Another interesting point of view for psychosis was presented back in 1986 by Robinson and Becker [56] who suggested that at least two distinct psychotic syndromes can result from chronic amphetamine administration. The first condition known as “amphetamine neurotoxicity” is caused by long-term exposure to increased amphetamine concentrations in the brain and is described by the term “hallucinatory-like” behavior and is associated with brain damage that results in a depletion of striatal dopamine and other brain monoamines. The second condition known as “behavioral sensitization” is caused by the administration of modest doses of amphetamine repeatedly and is defined as a gradual enhancement in numerous amphetamine-induced behaviors without causing brain damage or monoamine depletion.

Neuronal sensitization is a universal characteristic in the development of schizophrenia. Incentive learning is considered to be at the root of psychostimulant-induced context-dependent sensitization, which may play a role in the development of addiction, dyskinesia, and amphetamine-induced psychosis and it occurs when dopaminergic neurons are activated, for example by rewards. As a result, neutral stimuli gain incentive salience as well as the ability to trigger future attraction or other stimulus-seeking reactions [57]. Schmidt et al. suggested that if these pathological conditions arise as a result of a gradual process of sensitization, then the therapeutic effect should follow the principle of gradual desensitization. Thus, psychostimulant-induced sensitization and dopamine-deficiency-induced behavior deterioration follow a common pathobiological mechanism that proceeds in two opposite directions [57]. Although dopaminergic and glutamatergic neurotransmission have long been considered to modulate sensitization, the role of GABA-ergic metabolism and its influence on mesolimbic and mesocortical pathways, whose alterations are exhibited in schizophrenia, has received more attention lately [58] (Figure 2).

Although scientists declared glutamatergic neurotransmission as a targeted strategy for the development of treatments for schizophrenia 40 years ago [59,60], the pharmaceutical industry became interested in this direction only about a decade ago [61]. Early studies examined NMDA-receptor dysfunction through the lens of the widespread dopamine hypothesis of schizophrenia [62,63,64], while recent research is now focusing on NMDA-receptor deficiency as a primary element in dysfunctional brain networks leading to dopamine-mediated psychosis as a consequence [65,66,67].

#### 3.1.3. Schizophrenia as a Syndrome of Impaired Functional Brain Connectivity

The “dysconnection hypothesis” [68,69] describes schizophrenia as a disorder caused by a failure of functional integration in the brain and is based on a model of functional (synaptic) connectivity, specifically an abnormal regulation of synaptic efficacy. This hypothesis states that psychosis is best understood at a systems level, in terms of abnormal synaptic efficacy that mediates the effect of intrinsic and extrinsic connections. It suggests that the interactions between NMDA receptor activity and modulatory neurotransmitter systems are the fundamental pathophysiological substrate of schizophrenia. The core molecular mechanism that prevents individuals’ capacities to identify the right kinds of neuronal information for processing to generate coherent models of their world so they can understand it properly is a subtle but harmful failure of synaptic processing that mediates the functional integration or connectiveness of distributed brain processing [70].

Functional connectivity is a term that describes observable interactions between parts of the brain, but it does not specify how these connections are mediated and in what direction they interact. An effective connectivity, which is closer to the intuitive concept of connection, is utilized for a more thorough definition of the integration in the system (i.e., the influence that one neural system exhibits on another and the direction of interaction). In electrophysiology, there is a tight connection between effective connectivity and synaptic efficiency at the synaptic level [71].

Analyses of functional connectivity reveal various brain networks that represent distinct functions and diverse spatial topologies. Among the different brain networks that have been found to be malfunctioning in schizophrenia are the salience network (SN), the default mode network (DMN), and the central executive network (CEN), which together form the so-called “triple network” [72]. The SN consists of the anterior insula (AI) and the dorsal part of the anterior cingulum (dACC). By integrating sensory, emotional, and cognitive information, it engages in complex tasks including communication, social behavior, and self-awareness [73]. This network’s role is to control the dynamic changes in and between other networks, and it is essential for a rapid shift of focus. DMN is composed of the precuneus (PC), posterior cingulate cortex (PCC), medial prefrontal cortex (mPFC), and lateral parietal cortex. It activates when individuals concentrate on their inner experiences, such as dreaming, predicting the future, meditating, or recalling memories. DMN function is inversely correlated to brain networks that focus on external stimuli [74,75,76]. When an individual focuses their attention on a task, this network becomes deactivated [77]. CEN is composed of the dorsolateral prefrontal cortex (DLPFC), the posterior parietal cortex, the medial frontal gyrus (MFG), the superior frontal gyrus (SFG), the ACC, the paracingular gyrus, the ventrolateral prefrontal cortex (VLPFC), and subcortical areas such as the thalamus. This network is involved in executive functions, as well as coping with intentional, cognitively demanding activities [78], and intellectual ability control [79]. It is typically inactivated during rest [80,81,82] and plays a significant role in decision-making and active attention modulation [83].

**Figure 3:**
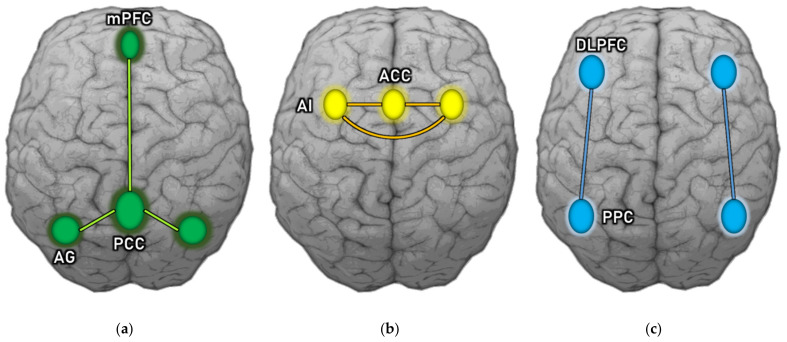
“Triple network”: (**a**) Default mode network; (**b**) Salience network; (**c**) Central executive network. AG—Angular gyrus; mPFC—medial prefrontal cortex; PCC—Posterior cingulate cortex; AI—Anterior insula; ACC—Anterior cingulate cortex; DLPFC—Dorsolateral prefrontal cortex; PPC—Posterior parietal cortex.

Impaired synchronization between the anti-correlated DMN and CEN is postulated as a key pathophysiological feature of schizophrenia [84]. The “triple network” is presented in Figure 3.

Table 1 presents the etiological hypotheses for schizophrenia, as well as some of the significant findings in support of each hypothesis.

#### 3.1.4. An Integral Etiological Framework for Schizophrenia

The concept that schizophrenia represents disintegration or fragmentation of the mind is as ancient as the term “schizophrenia” itself, which was coined by Bleuler [87] to highlight the “splitting” of the mind. Many of the fundamental symptoms (A-symptoms) presented by Bleuler, such as “associative disorganization,” emphasize the fragmentation and the lack of a continuous integration of the cognitive process. The nature of this integration (functional integration) at the neuronal level is an essential component in theoretical neurobiology [71].

The typical onset of schizophrenia in the transition phase between late adolescence and early adulthood is one of its main characteristics. Studies with rats demonstrate that ketamine and PCP are not neurotoxic until late adolescence. Furthermore, human investigations have shown that ketamine anesthesia does not cause psychotic symptoms in prepubertal children when compared to anesthesia in adults [85]. Olney et al. [86] suggest that this is due to a chain of neural connections involved in processes generating psychotic phenomena and neurotoxicity that result from NMDA-receptor antagonism, and this chain does not fully develop until the end of adolescence. According to Olney and Farber [50], impairment in the functioning of NMDA receptors (or the cells on which they are expressed) exists from the early stages of development, but the onset of psychotic symptoms occurs only after the full development of neural connections in early adulthood. This model supports the neurodevelopmental theory for schizophrenia, which speculates that brain abnormalities occur early in life, but remain dormant until late adolescence, when the pruning of neural connections occurs [88,89].

It has been suggested that glutamate may serve as a “bridge” uniting neurodevelopment and neurodegenerative theories due to its active participation in neuronal processes during all periods of human development. In early development, glutamate plays a role in neural migration, in adolescence it plays a role in the plasticity and pruning of neurons, and later in life, it is involved in neurodegeneration through the process of excitotoxicity [90].

Neurotransmitters tend to alter the excitability of neurons not only by directly affecting the postsynaptic membrane potential but also through modifying their responsiveness to other neurotransmitters. In the PFC, for example, dopaminergic terminal axons contribute to the development of synaptic complexes including excitatory (glutamatergic) projections on the pyramidal cells [91]. This synaptic arrangement represents the interplay of dopamine and glutamate neurotransmission, both of which are involved in modulating cortical connectivity. The brain connectivity is in a constant state of flow, where the neuronal dynamics influence the neuronal processes and the synaptic specializations. Therefore, connectivity is constantly changing (especially during growth). Brain plasticity (e.g., associative plasticity, self-organization, activity-dependent changes in synaptic efficacy, or experimentally induced long-term potentiation) combines processes such as brain connectivity and neuronal dynamics (i.e., interconnection). This is important because the “dysplastic” theories of schizophrenia and the theories of “impaired brain connectivity” are essentially the same and by default, both point to the hypothesis of impaired neurodevelopment.

In summary, schizophrenia can be considered as a mental disorder of impaired brain connectivity, the course of which is modeled by complex etiopathogenic factors, and at each stage of development, certain abnormal neurobiological processes contribute to its clinical manifestation. These processes originate from a genetic predisposition embedded in the genome, which is a prerequisite for the further development of the disease [92].

Later, during early development, the abnormalities stay dormant, followed by the prodromal phase, during which neuronal maturation occurs, then, due to endogenous neurochemical dysfunction and environmental factors, the full clinical manifestation of the disease occurs, reaching the residual phases of schizophrenia, during which neurodegenerative disorders predominate [93].

**Figure 4:**
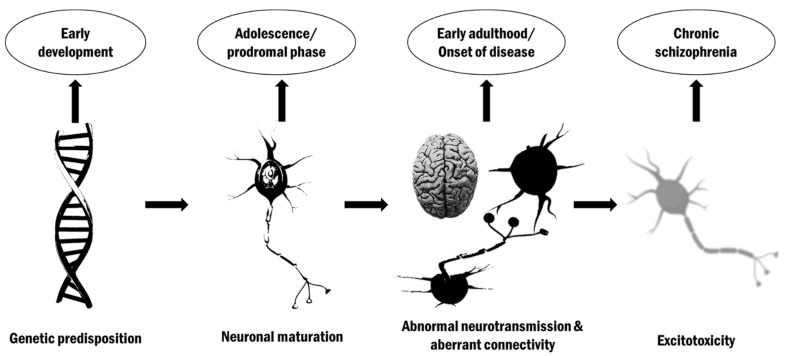
Integral etiological model of schizophrenia.

The integral model of schizophrenia is presented in Figure 4.

### 3.2. The Role of Neuroimaging and Translational Neuroscience in Schizophrenia Research

Understanding the complex dynamics of interactions between different brain areas at rest and during conditional task performance is a relevant subject in research about the underlying pathophysiological mechanisms of psychotic disorders. Disruptions in communication between and within brain networks, and the pathophysiological processes of their nodes enable translational neuroscience to obtain biological insight into schizophrenia and to describe it as impaired connectivity disorder [94].

A wide range of scientific fields and new methodologies are part of the translational approach in psychiatry. Magnetic resonance imaging (MRI) such as quantitative structural imaging, voxel-based neuromorphometry (VBM), functional neuroimaging, and spectroscopy are commonly used in research. Structural and functional MRI has the potential to refine diagnoses, assist in making therapeutic decisions, and be incorporated as a method of monitoring the effect of treatment by directly assessing the improvement of disease-related brain dysfunction. The different imaging modalities report diverse and specific CNS impairments, some of which will be presented in the following lines.

Table 2 presents important findings for schizophrenia, established by different modalities of magnetic resonance imaging.

Structural MRI investigations in psychotic individuals have revealed a variety of anatomical abnormalities in the CNS. Reduced GM volumes have been detected in several brain regions, including the frontal, temporal, parietal areas, the cingulate gyrus, and limbic structures (hippocampus, parahippocampus and thalamus) [95]. According to recent findings from the ENIGMA consortium, there is a considerable decrease in the size of the amygdala and a significant increase in the pallidum, which is directly associated with the longevity of the disorder [96] and can be interpreted as evidence in favor of the neurodegenerative hypothesis of schizophrenia. Brain abnormalities are also found in patients with their first psychotic episode, mainly as a reduction in GM volumes in the vermis, STG, operculum, etc. [97]. STG is a cortical zone that belongs to the auditory cortex, and the reduction in its GM volumes is associated with auditory hallucinations [112], which are a typical schizophrenic symptom. Another persistent finding is the enlarged lateral ventricles and the elevated cerebro-spinal fluid volume [98].

A recent study performed by our research group, consisting of a multivariate analysis of different MRI modalities, using artificial intelligence (unsupervised machine learning method) comparing paranoid syndrome in schizophrenia and depressive syndrome in the context of mood disorder, found that regions located in the left and right opercular part of the inferior frontal gyrus (IFG), right supramarginal gyrus, left STG, left anterior orbital gyrus, supplementary motor cortex, and several occipital areas are highly discriminatory for convergent cross-validation of biological features of disease [99].

One of the most consistent anatomical findings in schizophrenia is the abnormal insula structure. According to meta-analyses, the GM volume of the bilateral insula is reduced, and while evident in other mental illnesses, the reduced insula volume, is most pronounced in psychotic disorders [100,101,102]. This observation is present even in patients experiencing their first psychotic episode [103], and a progressive structural decrease is recorded throughout the course and chronicity of the condition [113].

fMRI research results collected during the performance of various tasks (task-related fMRI) while identifying activations in different brain areas when applying various sensory stimuli constitute a large part of the data published in the literature related to neuroimaging. Numerous task-related neuroimaging studies with emotionally charged visual stimuli have demonstrated reduced accuracy in recognizing emotions and prolonged response time in patients with schizophrenia [104,105,106].

In addition, task-related hyperactivation in the components of the DMN is observed in psychotic patients, while in healthy controls, deactivation in the same network is reported [107].

Studies have shown that impaired coordination of DMN/CEN/SN is associated with disorientation between internally and externally focused attention and cognitive impairment, which are typical signs of psychotic disorders [109]. Impaired synchronization between DMN and CEN results in an inability of the DMN to deactivate during cognitive load [108]. Zhou et al. [110], by conducting a fMRI study on 3 target groups (individuals with first psychotic episode without cognitive deficits, individuals with first psychotic episode with cognitive decline, and healthy controls), managed to prove that reduced task-related DMN suppression is a psychosis-specific biomarker for cognitive impairment, as the finding was established only in the group of psychotic individuals with cognitive decline.

Our research team, led by Stoyanov, has developed and integrated an innovative neuroimaging paradigm [108] aimed at translational cross-validation of von Zerssen’s Paranoid-Depressive Scale (PDS) [114] through its simulant implementation during functional imaging in individuals with paranoid and depressive syndrome. The obtained results confirm previous findings [107] that activations in the components of DMN in psychotic patients are completely absent among depressed patients. The novelty and distinctiveness of our design is that activations in DMN components are detected in schizophrenia patients during cognitive processing of paranoid-specific items on the scale. This discovery has a huge impact because it not only confirms prior findings from separate research, establishing a biomarker that is diagnostically specific for paranoid syndrome, but it also biologically validates von Zerssen’s PDS.

In this sense, psychiatry acquires a psychometric tool that is evidence-based and can be freely used in clinical practice. PDS can be implemented not only for initial diagnostic assessment, but also for monitoring the progress of the disease, as well as for monitoring the therapeutic effect, as the initial establishment of this scale was precisely in order to assess the effectiveness of treatment.

fMRI at rest (resting-state fMRI) examines the complex interactions between predefined ROIs using general linear model (GLM) and dynamic causal modeling (DCM) analysis. The study of functional connectivity at rest reveals a number of neural networks that represent specific patterns of synchronous activity [83]. Resting-state fMRI is used to identify dysfunctional integration or abnormal connectivity in the brain, either between individual brain regions or in between brain networks [115].

In healthy individuals, SN activation is often observed throughout a variety of cognitive tasks [116]. Its major purpose is to enable brain connectivity switching between the anti-correlated default mode and task-related states [117]. The insula is a key component of SN that executes a wide range of cognitive and affective functions, including self-awareness, emotional response and empathy.

Studies conducted by our team on resting-state effective connectivity prove an aberrant brain connectivity in schizophrenia [108,111], i.e., an inhibitory influence from PFC to Salience network (anterior insula) and an excitatory connection from the dACC to AI (hyperconnectivity of SN).

We suggest that the observed task-related hyperactivity of the DMN may be a consequence of the inhibition from the PFC on the insula, which disrupts its balancing function as a dynamic switch between the anti-correlated DMN and CEN.

In addition, the SN hyperconnectivity points to the conclusion that schizophrenic patients stay in a “resting-state of aberrant salience”, instead of a “resting-state of default mode”. Such a conceptual model helps understanding of schizophrenia as a behavioral disorder caused by disintegration in key brain networks. Abnormal SN hyperconnectivity and fronto-insular inhibition at rest prevent SN function from acting as a dynamic switch between resting-state (DMN) and event-related (CEN) activity. The fundamental disruption of SN in schizophrenia prevents switching between anti-correlated DMN and CEN, thus interfering with their basic functions.

**Figure 5:**
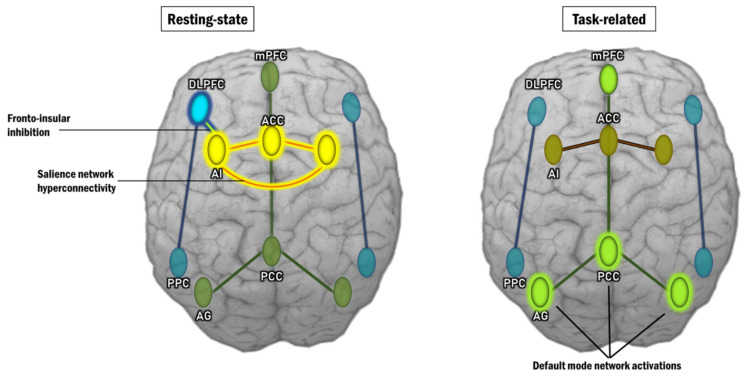
Resting-state aberrant connectivity and task-related abnormal activations that are observed in schizophrenic patients. AG—Angular gyrus; mPFC—Medial prefrontal cortex; PCC—Posterior cingulate cortex; AI—Anterior insula; ACC—Anterior cingulate cortex; DLPFC—Dorsolateral prefrontal cortex; PPC—Posterior parietal cortex.

This concept is presented in Figure 5.

### 3.3. Implications of Neuroimaging Findings for the Treatment of Schizophrenia

Given the controversial efficacy of standard therapies (psychopharmacology and psychotherapy), a significant proportion of schizophrenic patients do not achieve full remission (i.e., become asymptomatic) or maintain symptom relief. This lack of efficacy has prompted the search for alternate treatments, which include the use of more invasive procedures for treatment-resistant cases of schizophrenia [118]. As a result, the focus has shifted to methods for neuromodulation or modification of connections between different pathways and neurotransmitter systems in the brain. This interest is motivated by the improved neurobiological views of the origin of psychosis and the neuroimaging findings, which have led to the ability to regulate the central disruptions through direct and focal modulation of brain activity [94].

Unconventional therapeutic instrumental methods have been shown to be beneficial for a variety of psychiatric disorders (e.g., electro-convulsive therapy for depression, mania, and catatonia; transcranial magnetic stimulation for treatment-resistant depression; transcranial direct current stimulation for cognitive deficiency in schizophrenia; deep brain stimulation for obsessive-compulsive disorder, addiction, and severe forms of depression). Unfortunately, their usage for schizophrenia is still limited in standard clinical protocols, and these technologies remain in the shadow of psychopharmacology [94].

However, we remain positive about the possibilities for improving the therapeutic approach to psychosis, as some of the modern neuroimaging techniques can provide available resources for non-invasive analysis of complex interactions and biochemical imbalances that cause the characteristic manifestation of the disease. The study of the pharmacokinetic and pharmaco-dynamic processes occurring in the CNS under the load of various chemicals could help both to identify psychosis-specific biomarkers and to develop an effective strategy for monitoring and predicting the effect of drug treatment.

## 4. Discussion

One of the most essential elements of comprehending inter-individual differences or clinical results is to investigate the neurochemical substrates of brain function. Positron emission tomography (PET) has allowed for a direct assessment of brain chemistry in vivo. The dynamics of regional uptake of neurotransmitter-specific radio-ligands in PET can be used to identify regional neurochemical modulation of brain activity. PET mostly uses 18FDG, a direct reflection of regional glutamate transmission, to offer a quantifiable measure of glucose metabolism [119]. PET is considered as a “gold standard” for identification of chemical imbalances in the brain and has been investigating for a long time the neural correlates of psychiatric conditions like exogenous psychosis caused by ketamine [120,121], depression [122,123] and euphoric intoxication [124]. The downfall of PET is that it is an expensive tool that operates with specific ligands, and it is not appropriate for repeated research due to the dose-radiation restrictions protocols, with some Ethics commissions completely forbidding it in healthy individuals. Furthermore, it can capture a single “snapshot” binding to a receptor molecule, within the limited time between administration and semi-elimination of the radio-ligand.

Therefore, the focus is on pharmacological MRI (ph-MRI), which is an accessible instrument as an alternative for studying the chemical imbalances in the brain. ph-MRI is an innovative technology for assessing regional network effects and treatment response to specific medications. In general, there are two types of ph-MRI. The first is usually done as a drug challenge study, in which MRI signal changes are evaluated after an acute administration of the substance of interest. Apparently, there are several adaptations on this fundamental paradigm, such as drug antagonistic effects or investigating the acute effects of one medication on the chronic effects of another (useful perhaps for studying drug addictions). The second type of ph-MRI is the observation of pharmaco-modulatory effects of drugs on a traditional task-related fMRI study, such as dopaminergic medicines’ effects on cognitive tasks [125].

The main purpose of phMRI is to recognize the location of drug action fingerprinting in order to measure the connection between drug dose, neural reaction, and treatment significance over time (pharmacokinetic/pharmacodynamic modeling), and to assist in making go–nogo decisions about the efficacy of drug treatment in clinical trials, all with the goal of accelerating drug discovery. By enabling a controlled regulation of a specific pathway and analyzing its causal influence on other signals and systems, pharmacological probing tests can be useful for fundamental neuroscience and validation studies [119].

The dopamine and glutamate neurotransmitter systems are the main targets for ph-MRI in schizophrenia. Suitable ligands for examining the dopamine system are cocaine, amphetamine, and *L*-DOPA, while ketamine, phencyclidine (PCP), and LY2140023 are appropriate substances for studying the glutamate systems [125]. The main advantage of ph-MRI is that it can investigate the effects of pharmacological agents at the network level and remotely from areas of high target receptor densities, whereas PET and molecular studies can define target receptor occupancy and affinity without necessarily translating effects to large-scale networks [126]. As a result, ph-MRI provides a “system evaluation” of networks underpinning a drug’s behavioral effects, irrespective of its pharmacological mechanism of action [127]. Because functional MRI can monitor the cumulative effect of these interactions across many brain areas, ph-MRI has the potential to generate “mechanism-related activation maps” that may be used as targets for drug testing [128].

Given the intricacy of the clinical presentation and the underlying malfunctioning brain circuits, ph-MRI investigations can assist in determining the initial treatment response, mechanisms of therapeutic efficacy and adverse effects, and potentially accelerate CNS drug development. ph-MRI studies reveal stable and reproducible alterations on disease-relevant networks, as well as sensitivity to early pharmacological impacts on disease-related functional architecture. Improved disease phenotyping, or biomarkers, utilizing sophisticated imaging techniques will substantially assist future CNS medication research and development.

## 5. Conclusions

Schizophrenia and other psychotic disorders affect a huge number of people around the world, thus destroying the lives of patients, burdening their loved ones and society, and leading to significant and global economic losses. Theories about the origin of schizophrenia have changed over the years, starting with the supernatural, going through the psychodynamic “schizophrenogenic mother” and reaching modern interpretations of the etiology of this destructive disease. For more than half a century, neurobiology has been able to provide explanations for the central brain mechanisms of the disease through the neurodevelopment hypothesis, the neurodegeneration hypothesis, and the biochemical dysfunction represented by the dopamine and glutamate hypotheses. In recent decades, thanks to neuroimaging, new insights have been gained about the disrupted integration in communication between and within the brain networks, emphasizing the disrupted connectivity between the components of the triple network, the default mode network, salience network and central executive network, which form the concept of schizophrenia as a brain dysconnectivity syndrome. At this stage, neuroscience has the task of combining different concepts into an integral model with the clear goal of contributing to the development of new treatments for schizophrenia. A suitable tool for this is pharmacological magnetic resonance imaging which has the ability to measure the connection between drug dose, neural reaction, and treatment significance over time, thus providing a “system evaluation” of networks underpinning a drug’s behavioral effects, irrespective of its pharmacological mechanism of action.

According to the validation theory as conceived back in 2007, the cross-validation of clinical psychological self-assessment tools with fMRI may well underpin pharmaco-psychological monitoring strategies [129].

Advances in neuroscience are expected to provide new horizons in the near future by incorporating neuroimaging findings into clinical practice, including non-traditional neuromodulation methods in the therapeutic approach of schizophrenia.

## Figures and Tables

**Table 1:** Etiological frameworks of psychosis.

Concept	Definition	Studies	Main Findings/Conclusion
Dopamine hypothesis	Hyperdopaminergia in the mesolimbic system causes psychotic symptoms and hypodopaminergia in the mesocortical pathway is the reason for negative symptoms.	Seeman and Lee (1975) [36]	Neuroleptics’ efficacy in blocking dopamine D2 receptors.
		Lieberman et al. (1987) [37]	Psychotic phenomena are triggered by the dopamine agonists.
		Breier et al. (1997) [38] Laruelle et al. (1999) [39] Abi-Dargham et al. (1998) [40] Kegeles et al. (2000) [41]	Amphetamine-induced hyperdopaminergia in the striatum is significantly higher in patients with schizophrenia compared to healthy controls.
		Gibbs (1951) [42]	Epileptic seizures caused by lesions in the limbic regions lead to florid psychotic production.
		Malamud (1967) [43]	Tumors in the limbic structures cause psychotic symptoms.
Glutamate hypothesis	A decrease of glutamate activity at the glutamate synapse, particularly in the prefrontal cortex induces positive, negative, and cognitive symptoms that are virtually indistinguishable from those seen in schizophrenia.	Kegeles et al. (2000) [41]	Amphetamine-induced hyperdopaminergic activity in schizophrenia result from disturbances in the glutamatergic neuronal systems that regulate dopaminergic cellular activity.
		Lahti et al. (1995) [46] Kapur et Seeman (2002) [47] Frohlich and Van Horn (2014) [48] Javitt (2002) [49]	Phencyclidine and ketamine which are dissociative anesthetics act as glutamate antagonists by blocking the glutamate receptor of the *N*-methyl-*D*-aspartate (NMDA) type and induce positive, negative, and cognitive symptoms.
		Olney and Farber (1995) [50] Grace (1991) [51]	Impaired dopaminergic neurotransmission in schizophrenia may itself be secondary to the abnormal NMDA-receptor neurotransmission.
		Goldman-Rakic and Selemon (1997) [52] Weinberger and Berman (1996) [53] Du et al. (2019) [54] Walton et al. (2018) [55]	Abnormalities of glutamatergic afferent neurons from the prefrontal cortex to the dopaminergic subcortical areas of the midbrain are associated with abnormal dopamine regulation.
		Coyle (1996) [66] Lisman et al. (2008) [67]	NMDA-receptor deficiency may be the primary element in a dysfunctional brain network leading to dopamine-mediated psychosis in consequence.
		Stone et al. (2007) [85]	Ketamine anesthesia does not cause psychotic symptoms in prepubertal children when compared to anesthesia in adults (onset of schizophrenia in early adulthood).
		Olney et al. (1999) [86]	A chain of neural connections involved in processes generating psychotic phenomena and neurotoxicity result from NMDA-receptor antagonism, and this chain does not fully develop until the end of adolescence (onset of schizophrenia).
Dysconnection hypothesis	Schizophrenia can be described as impaired connectivity disorder caused by a failure of functional integration in the brain and is based on a model of functional (synaptic) connectivity, specifically an abnormal regulation of synaptic efficacy.	Robinson and Becker (1986) [56] Schmidt and Beninger (2006) [57]	Incentive learning is thought to underpin psychostimulant-induced context-dependent sensitization, which may be important in the development of addiction, dyskinesia, and amphetamine-induced psychosis.
		Bolton et al. (2020) [72]	The “triple network” system is malfunctioning in schizophrenia.
		Williamson (2007) [84]	Impaired synchronization between the anti-correlated Default mode network and Central executive network is a key pathophysiological feature of schizophrenia.

**Table 2:** Magnetic resonance imaging findings in schizophrenia research.

Magnetic-Resonance Imaging Technique	Study	Main Findings/Conclusion
Structural neuroimaging	Zhuo et al. (2017) [95]	Schizophrenic patients have reduced gray matter volumes in the frontal, temporal, and parietal areas, the cingulate gyrus, and limbic structures (hippocampus, parahippocampus and thalamus).
	van Erp et al. (2016) [96]	Decrease in the amygdala and increase in the pallidum, which is directly associated to the longevity of the disorder which can be interpreted as evidence in favor of the neurodegenerative hypothesis of schizophrenia.
	Chang et al. (2016) [97]	Reduction in grey matter volumes in the vermis, superior temporal gyrus, operculum.
	Wright et al. (2000) [98]	Elevated cerebro-spinal fluid volume.
	Stoyanov et al. (2021) [99]	Regions located in the left and right opercular part of inferior frontal gyrus, right supramarginal gyrus, left superior temporal gyrus, left anterior orbital gyrus, supplementary motor cortex, and several occipital areas are highly discriminatory for convergent cross-validation of biological features of psychosis vs. depression.
	Shepherd et al. (2012) [100] Goodkind et al. (2015) [101] Sheffield et al. (2020) [102] Lee et al. (2016) [103] Mier et al. (2014) [104]	Grey matter volume of the bilateral insula is reduced in psychotic disorders, and a progressive structural decrease is recorded throughout the course and chronicity of the condition.
Functional task-related neuroimaging	Mier et al. (2014) [104] Goghari et al. (2017) [105] Belge et al. (2017) [106]	Reduced accuracy in recognizing emotions and prolonged response time in patients with schizophrenia.
	Whitfield-Gabrieli et al. (2009) [107]	Task-related hyperactivation in the components of the Default mode network psychotic in patients, while in healthy controls–deactivation in the same network.
	Stoyanov et al. (2021) [108]	Activations in Default mode network components during cognitive processing of paranoid-specific items from the von Zerssen’s Paranoid-depressive scale [108].
Functional resting-state neuroimaging	Nekovarova et al. (2014) [109]	Impaired coordination of Default mode network / Central executive network / Salience network is associated with disorientation between internally and externally focused attention and cognitive impairment.
	Zhou et al. (2016) [110]	Reduced task-related Default mode network suppression is a psychosis-specific biomarker for cognitive impairment, as the finding is established only in psychotic individuals with cognitive decline.
	Stoyanov et al. (2021) [108] Aryutova et al. (2021) [111]	There is a strong aberrant brain connectivity in schizophrenia–an inhibitory influence from prefrontal cortex to Salience network (anterior insula) and an excitatory connection from the anterior cingulate cortex to anterior insula [108,111].
